# Revolution or Evolution? Technical Requirements and Considerations towards 6G Mobile Communications

**DOI:** 10.3390/s22030762

**Published:** 2022-01-20

**Authors:** Saddam Alraih, Ibraheem Shayea, Mehran Behjati, Rosdiadee Nordin, Nor Fadzilah Abdullah, Asma’ Abu-Samah, Dalia Nandi

**Affiliations:** 1 Department of Electrical, Electronic and Systems Engineering, Universiti Kebangsaan Malaysia, Bangi 43600, Selangor, Malaysia; eng.alraih@gmail.com (S.A.); mehran.behjati@ukm.edu.my (M.B.); fadzilah.abdullah@ukm.edu.my (N.F.A.); asma@ukm.edu.my (A.A.-S.); 2 Electronics and Communication Engineering Department, Faculty of Electrical and Electronics Engineering, Istanbul Technical University, Istanbul 34467, Turkey; ibr.shayea@gmail.com or; 3 Indian Institute of Information Technology (IIIT), Kalyani 741235, India; daliadas311@gmail.com

**Keywords:** 6G, beyond 5G, future networks, next-generation mobile, terahertz, VLC, wireless transmissions

## Abstract

Ever since the introduction of fifth generation (5G) mobile communications, the mobile telecommunications industry has been debating whether 5G is an “evolution” or “revolution” from the previous legacy mobile networks, but now that 5G has been commercially available for the past few years, the research direction has recently shifted towards the upcoming generation of mobile communication system, known as the sixth generation (6G), which is expected to drastically provide significant and evolutionary, if not revolutionary, improvements in mobile networks. The promise of extremely high data rates (in terabits), artificial intelligence (AI), ultra-low latency, near-zero/low energy, and immense connected devices is expected to enhance the connectivity, sustainability, and trustworthiness and provide some new services, such as truly immersive “extended reality” (XR), high-fidelity mobile hologram, and a new generation of entertainment. Sixth generation and its vision are still under research and open for developers and researchers to establish and develop their directions to realize future 6G technology, which is expected to be ready as early as 2028. This paper reviews 6G mobile technology, including its vision, requirements, enabling technologies, and challenges. Meanwhile, a total of 11 communication technologies, including terahertz (THz) communication, visible light communication (VLC), multiple access, coding, cell-free massive multiple-input multiple-output (CF-mMIMO) zero-energy interface, intelligent reflecting surface (IRS), and infusion of AI/machine learning (ML) in wireless transmission techniques, are presented. Moreover, this paper compares 5G and 6G in terms of services, key technologies, and enabling communications techniques. Finally, it discusses the crucial future directions and technology developments in 6G.

## 1. Introduction

The rapid growth of smart emerging technologies and their features (e.g., real-time, interactive services) have contributed to the significant increase in wireless data traffic that cannot be fully supported sufficiently by the current network, even with fifth generation (5G) systems [1]. That indicates that the available deployed mobile networks will not be able to fully and efficiently keep up with the changing technical standards. Furthermore, a more developed digital society that is driven by nearly instantaneous and limitless wireless connectivity by 2030 is projected [2]. This has propelled the development of sixth generation (6G) to meet high technical standards of new spectrum- and energy-efficient transmission methods [1,3].

The 6G mobile networks are expected to support a highly dense network connecting 500 billion machines [4]. Its capacity can go up to 1000 times higher than the capacity of 5G. Through the 6G mobile network with a higher frequency spectrum, users can expect an even higher speed (where the data transfer rate is expected to be 100 to 1000 times higher than what 5G offers), enhanced capacity, and even lower latency that can support the prospects of new applications, including precision medicine, intelligence disaster prediction, and virtual reality (VR). In particular, 6G would offer data transfer rate in Gbps to Tbps through the utilization of multi-band high-spread spectrum that includes 1–3 GHz band, millimeter wave (mmWave) band (30–300 GHz), and terahertz (THz) band (0.06–10 THz) [5,6].

Considering the development trend of earlier mobile networks, the initial 6G mobile network would adopt the current architecture and strengths of 5G, such as higher frequency bands and an optimized decentralized network system. Although 6G mobile technology will provide several advantages to future ultra-dense networks, there are several issues that will still need to be highlighted, discussed, and then addressed before the standardization of the 6G system. Therefore, conducting a survey paper that considers different aspects and discusses technical challenges from different perspectives will contribute to achieving these targets. In the literature, there are plenty of reputable 6G review journals and white papers that have been published. There are hundreds of survey papers related to 6G technology, but in this paper, only a few reputable articles are selected to be discussed and their limitations to be highlighted, as summarized in the following paragraphs.

The research paper of [7], presents an extensive survey of the existing developments regarding 6G. In addition, it shows the societal and technological trends that are leading towards the 6G movement. After that, the latest applications that comprehend the demands caused by 6G driving trends are explained. In addition, it discusses the requirements needed to attain the 6G applications. The article then presents a detailed description of the critical enabling technologies and a brief overview of the existing research projects and activities, including standardization efforts towards 6G development. Finally, a summary of the lessons acquired from the latest research is presented, and the technical difficulties faced are elaborated, which would present the directions for future research in the field of 6G.

A review study in [8] is also another extensive survey work conducted and focused on the evolution of wireless technology towards 6G networks. The focus is mainly on the latest architectural modifications related to 6G networks, defined by universal three-dimensional (3D) coverage, the inception of pervasive artificial intelligence (AI), and improved network protocol stack. In addition, the authors also discuss the relevant aspiring technologies that help create sustainable and socially impeccable networks, involving THz and visible light communication (VLC), blockchain, new communication paradigms, and symbiotic radio. As asserted by the authors, the objective of the study is to offer increased guidance to facilitate future research in the field of green 6G.

Reference [9] presents a thorough discussion on 6G on the basis of the review of 5G developments, including visions and requirements, technological developments, and challenges. The objective is to address the issues of capacity, coverage, and user data rate as well as the movement speed of mobile communication systems.

An alternative survey study is also conducted in [10]. The paper discusses the most aspiring research areas from the latest literature that have a common direction for the 6G projects. It mainly contributes towards examining the vital issues and possible attributes of 6G communications, such as (i) vision and main features, (ii) challenges and possible solutions, and (iii) research activities. An extensive examination of these disputed research topics was performed with respect to the motivation of their distinct sub-domains so that a clear, concrete, and succinct conclusion could be made.

Subsequent review work is also conducted in [11] that considered a distinct review approach. The work presents and discusses a sample of “Internet of things” (IoT) use cases that indicate the different types of its implementations. The use cases are selected from the most research-active segments that may take advantage of 6G and its enabling technologies. These include the domains of healthcare, transport, smart grids, and Industry 4.0. Furthermore, a few of the practical issues encountered and the lessons learned from implementing these use cases are determined. In this review, the cases’ key requirements are discussed as well as how they overlap with the main drivers for the upcoming generation of wireless networks.

A historical review study of past networking technologies and the way they have determined the existing trends in 6G networking was first presented in [12]. After this, it elaborates on the four main areas of 6G networks: distributed AI, real-time intelligent edge computing, 3D intercoms, and intelligent radio. In addition, it also elaborates on certain potential development technologies in each domain in addition to the related security and privacy challenges that emerge. A report on the possible application of 6G is finally presented in the survey.

In addition to this, a critical assessment of the vision of 6G wireless communication and its network structure is carried out in the survey study [13]. The study presents an overview of the various critical technical challenges experienced, certain potential solutions relevant to fourth generation (4G), the physical layer transmission processes, network designs, and security measures.

Although every paper has outlined several contributions, no comprehensive paper addresses technical challenges, highlighting potential applications and key enabler transmission technologies and techniques. Therefore, this paper aims to provide an additional and different contribution to the literature, as a survey paper, compared with the other 6G papers that have been selected and discussed previously.

The overview study examines the key improvements provided by 6G technology and compares them with the previous mobile technologies. That includes the data rate speed, system capacity, latency, and other key improvements, such as supporting very high mobility optimal coverage, mobile edge computing, enabling augmented reality (AR), and connection reliability. The paper also discusses the key enabler technologies that will be the main factor for enabling 6G technology to meet the drawn targets. That covers the spectrum of communication technologies, such as THz communication and VLC. The key technical challenges and future trends are also discussed and highlighted. Moreover, this paper compares 5G and 6G in terms of services, technologies, and enabling key technologies.

The remainder of the paper is structured as follows: Section 2 summarizes the evolution of mobile wireless communication from first generation (1G) to 6G. Section 3 presents 6G use cases and applications. Next, Section 4 discusses 6G requirements. Subsequently, 6G radio frequency (RF) spectrum technologies are presented in Section 5. After that, Section 6 provides key enabling technologies for 6G and relevant preparation and implementation. Next, several challenges of 6G are demonstrated in Section 7. Subsequently, future directions in 6G technology development are presented in Section 8. Finally, Section 9 concludes the review paper.

## 2. Evolution of Mobile Communication

In this era of wireless communication networks, the mobile communication industry, particularly its transmission technology and frequency bands, has demonstrated outstanding growth from the mobile system to the upcoming 6G mobile system. Every generation has its specific attributes, systems, and potentials [14].

The 1G mobile network, which relies on analog transmission for speech services, started in the earlier 1980s. Back in 1979 was the first launch of the Nippon Telephone and Telegraph (NTT) cellular system provider, which started in Tokyo, Japan. Then, two years later, Europe launched its cellular system. Total Access Communication Systems (TACS), and Nordic Mobile Telephones (NMT) were the most well-known analog systems [15]. Although 1G was the first mobile system, it caused a revolution in the mobile system technologies at that time. It used an analog transmission signal which supported only voice services with a minimum data rate of up to 2.4 kbps. Additionally, it operated at a frequency range of 800–900 MHz with a bandwidth of 40 MHz and 30 kHz of channel capacity. First generation used frequency division multiplexing (FDM). Since 1G used the analog signal for transmission, there were some problems, such as low-quality calls, high energy consumption, and poor data capacity security [16].

Following that, in 1991 the second generation (2G) mobile network was introduced. Conceptually, this mobile network involves a uniform distribution of several base stations (BSs) globally to provide multiple access points (i.e., FDMA, code-division multiple access (CDMA), and time-division multiple access (TDMA)) [17] for users to communicate with one another. Accordingly, 2G technologies, such as 2G Global System for Mobile Communications (GSM), 2.5G General Packet Radio Service (GPRS), and 2.75G Enhanced Data Rates for Global Evolution (EDGE), rely on the compression–decompression algorithm (codec) [18]. Compared to 1G, 2G provided some more services, such as “short message service” (SMS) and “multi-media service” (MMS), and it provided better quality in services. In addition, 2G was upgraded to work in the frequency range of 850–1900 MHz and data rate up to 64kbps. Second generation used TDMA and CDMA. Overall, 2G gave a better mobile communication service and encrypted data transmission using digital technology [19].

The mobile network continued to develop with the introduction of the third generation (3G). Third generation involves technical standards of IMT-2000 that incorporate elements of reliability and speed, specifically a data transfer rate of at least 200 kpbs [20]. With higher connection speed, the network transformed from a conventional mobile network to portable media devices (e.g., computers, gaming consoles, and tablets). Apart from quality internet services, 3G offers improved security through user authentication features when users connect to other wireless devices [17]. Accordingly, three fundamental technologies for the 3G network are CDMA2000, Wideband Code Division Multiple Access (WCDMA), and Time Division Synchronous Code Division Multiple Access (TD-SCDMA).

Then, 4G was introduced after 3G, where users could access the network anytime and anywhere. Fourth generation is synonymous with the term “MAGIC”, which refers to “mobile multimedia anywhere, global mobility solutions over integrated wireless and customized services” [14]. Through 4G, users are able to experience smooth network access and end-to-end IP transmission as well as quality of service (QoS) management with higher service quality, mobility, and a data transfer rate of 20 Mbps.

The commercial application of 5G started back in 2019, after the initial complete set of 5G standards was finalized. The introduction of 5G marks the start of a global digital era with revolutionary wireless technology standards, particularly in terms of data transfer rate, latency, mobility, and even the number of connected devices [21]. Users of 5G can expect tremendous improvements, such as a data transfer rate of up to 10 Gbps, significantly lower latency (at almost 10 ms) at a higher capacity, reliability, and QoS [22,23]. The features of 5G have truly set this advanced mobile network apart from its predecessors. Unlike its predecessors, 5G is the first to utilize the mmWave band, a new technology of frequency band. Besides that, 5G is the focal infrastructure of IoT and is capable of integrating numerous new technologies, such as device-to-device (D2D) communication, software-defined networking (SDN), and massive multiple-input multiple-output (mMIMO) [24,25,26,27].

The 5G signifies the revolutionary development of communication networks. Using a single platform, 5G offers various services, from advanced mobile broadband communications, automated driving, and VR to the IoT. With the recent decade’s rapid technological advancements and innovations, going beyond 5G to satisfy the growing technological needs and demands at all levels is inevitable.

The year 2030 is primarily targeted to be the year of launching the first commercializing system for 6G technology. A global digital society that is highly driven by highly advanced and almost instantaneous wireless connectivity by 2030 is envisioned [28,29]. For this, 6G will play a central role in integrating seamless wireless connectivity and various technology functions (e.g., caching, communication, computing, control, imaging, navigation, positioning, radar, and sensing) that support full-vertical applications. Basically, 6G is a self-governing machine that can imitate human intelligence and consciousness and offers various means of communication and interaction with smart terminals (e.g., through brainwaves or neural signals, eyes, fingers, and voice).

As a vision for the future, and due to 6G potentially utilizing a very high spectrum as compared to previous generations [30], 6G will dramatically improve the data rate speed, up to 100–1000 times faster than 5G [5]. Therefore, hundreds of Gbps to Tbps links will be available in 6G networks using the multi-band high-spread spectrum [31]. For instance, the combination uses 1–3 GHz band, 30–300 GHz band in mmWave, and 0.06–10 THz band in THz. Moreover, in terms of capacity, 6G aims to sharply increase the capacity by up to 1000 times more than 5G. For example, upper trillion-level objects will be connected to 6G, whereas billion-level mobile devices connect to 5G. Also, in terms of latency, 6G will provide latency up to 10–100 μs. In other words, the evolution of the mobile communication networks from 2G to 5G was centered on servicing people, which means the latency depended on human reaction times, such as the visual reaction time (~10 ms), the auditory reaction time (~100 ms), and the perceptual response time (~1 ms) [3].

Sixth generation is expected to significantly change wireless evolution from connected things to connected intelligence. Furthermore, 6G is required to support ubiquitous AI services from the network’s core to the end devices [10]. In other words, AI will be an essential factor in designing and optimizing protocols, the architectures, and the operation of 6G [9,32].

## 3. Use Cases and Applications of 6G

This section presents some of the potential use cases and applications of 6G for industry and public consumers. Fifth generation is evolving, and what has been deployed in 5G mainly focused on mobile broadband, while other aspects of 5G, such as automation, low latency, and high reliability for manufacturing, e-health, gaming, and connected vehicles, are yet to come and are subject to the Release 16, 17, and 18 [33,34,35]. The network technology is evolving to a flexible and agile infrastructure, and it is expected that 6G, like the previous generations, will be influenced by today’s emerging technologies.

“Twelve Global Megatrends” key themes are expected to shape society at large by 2030, as proposed by Frost & Sullivan’s Visionary Innovation Group [36]. The 6G network will satisfy the requirements of many forthcoming technological trends and themes, such as factories of the future, autonomous mobility, and towards “zero” world (“zero” energy, touch, and error).

So far, communication systems have transmitted data that interact with the two human senses, hearing and seeing. Thus, one of the main goals of 6G is to transmit data related to other human senses, such as touch, smell, and taste. This additional human sense transmission refers to the fourth dimension (4D), which will allow the communication systems to transmit other sensory information, such as air, noise, and light pollution. In this regard, 6G can be broadly characterized as integration between communication and sensing, which would lead to much smarter, relevant, and personalized services and would effectively and naturally integrate the physical and virtual world together [37].

Sixth generation is also going to use mobile technology to deliver AI to everyone, anywhere, at any time, that will leverage the 6G wireless network as a sensor network. Therefore, by integrating communication with sensing and AI, 6G will be an intelligent network that will open and unlock several new industrial applications, such as intelligent video analysis, real-time ubiquitous intelligence systems, and intelligent customer engagement services. The following points list some of the potential applications of 6G [38,39]:

- Transportation: autonomous vehicles interacting with the environment and cooperating driving.
- Retail: near real-time and end-to-end no-touch transactions from production to consumption.
- Manufacturing: autonomous and dynamic reconfigurable production lines based upon near real-time demand.
- Healthcare: robotic controlled surgery, remote surgical procedures, and enhanced precision surgical capabilities.
- Industrial/Public: cooperative manufacturing, massive embedded intelligence, low-power sensor deployment across supply chain and surroundings.
- High fidelity gaming: Play instantly without download, subscription enabled, and direct distribution model.
- Robotic construction: autonomous worksite equipment, real-time on-site planning and construction.
- Instantaneous delivery: massive fleet logistics and dynamic delivery locations.
- Autonomation: process autonomation, cognitive process autonomation, virtual agents and analytics, AI- and machine learning (ML)-based execution, and intelligent enterprise.
- Truly immersive extended reality (XR): Combining VR, AR, and mixed reality (MR) and utilizing it in entertainment, medicine, science, and education.
- High-fidelity mobile hologram: as a next-generation of media technology to provide real-time services.

In 6G, ultra-reliable low latency communications (uRLLC), enhanced mobile broadband (eMBB), and massive machine-type communication (mMTC) will be extended to another three dimensions [40], which are ubiquitous mobile ultra-broadband (uMUB), ultra-high data density (uHDD), and ultra-high-speed-with-low-latency communications (uHSLLC). Meanwhile, uMUB in 6G systems enables the transmission of any desired performance throughout the space-aerial-terrestrial-sea domain. While uHSLLC provides ultra-high speeds and very low latency, uHDD meets the criteria for data density and excellent dependability. Additionally, upcoming uMUB, uHDD services, and uHSLLC need end-to-end co-design of communication, sensing, and computing capabilities.

In order to enhance 5G use cases, three new use cases are upgraded, as 5.5G, to the main three 5G use cases, which are real-time broadband communication (RTBC), uplink centric broadband communication (UCBC), and harmonized communication and sensing (HCS) [41]. As Huawei’s vision and mission, these three cases will lead beyond the Internet of everything. (IoE), enabling intelligent IoE.

**Figure 1:**
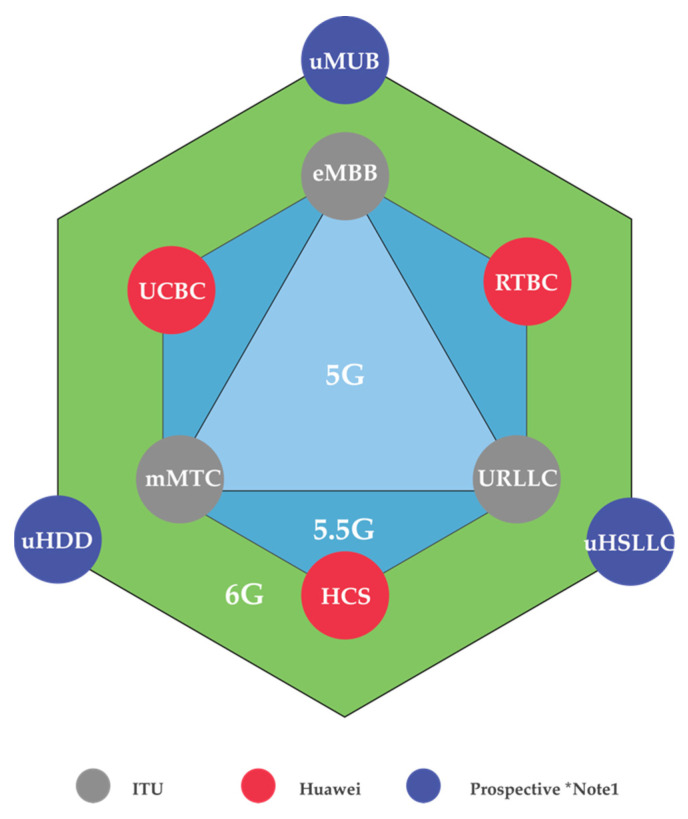
Comparisons of use cases for 5G and 6G systems. * Note 1: Ref. [40].

The comparison of the key scenarios of 5G, 5.5G, and 6G is shown in Figure 1. Sixth generation is expected to inherit some of the features and technologies of 5G, evolve some of the current transmission technologies based on future demands, and revolutionize some technologies based on the demanded use cases.

To realize the above applications on a large scale, a network with extremely high data rate, reliability, and power efficiency is required. The next section considers the 6G requirements in detail.

## 4. Requirements of 6G

To compare 6G with 5G, the focus of 5G is more on speed, mass connectivity, reliability, and latency, while the suggested focus directions for 6G are sustainability, coverage connectivity, synchronization, geo-location, and trustworthiness [37].

Fifth generation is capable of a low latency of one millisecond, while it was not designed with guaranteed time synchronization. Therefore, there is a time difference between different flows or different sessions generated by different objects that need interactions with each other. In 6G, multiple objects will integrate with each other, either in virtual or physical worlds. Therefore, the time differences between all objects and the communication links need to be within a certain limit to provide natural reaction and interaction between them. On the other hand, time jitter is also highly important for future communications which are beyond 5G capability. As the objects’ interaction in 6G is essential, the need for high-quality and high-resolution geo-location plays a vital role in realizing 6G.

Connectivity is another critical problem of 6G that should be addressed properly. The future network not only needs ultra-high-speed connectivity but also needs resilience and adaptive and customizable connectivity. For example, for 4D video transmission, a large amount of data needs to be transferred, in the range of terabits per second; therefore, along with a high bandwidth, smart algorithms are required to compress data, for example, with the help of AI to predict the next posture of a face.

On the other hand, such exhaustive data transmission needs massive backhaul with low latency. In addition, 6G requires flexible spectrum access, high-resolution channel state information (CSI), and precoding for distributed mMIMO, high-capacity uplink, higher network capacity and quality for mobile cloud XR, and low-power, high-precision positioning [42,43,44]. Another connectivity target of 6G is to overcome the digital divides, where everybody, irrespective of their location, has decent access to the network services.

On another extreme, massive low-powered devices or “things” communication requires resource utilization for high connection density but which also supports a timely, efficient, and reliable mechanism to deliver data. The 6G network would provide a very high peak transmission rate, up to 1–10 Tbps, with very low latency, as low as 10–100 μs, but all of this needs to be achieved while having much lower energy consumption not only per unit data but also as overall power consumption [45].

**Figure 2:**
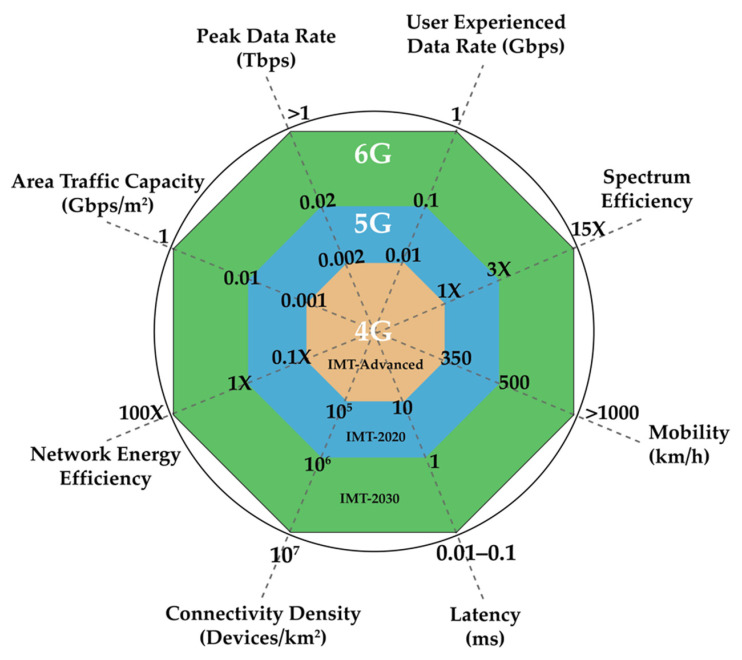
Key capabilities of 6G networks [48].

Overall, 6G offers remarkable network capabilities that are imperative for realizing the 2030 intelligent information society. Figure 2 presents the technical requirements of 6G, compared with 4G and 5G requirements, which include the following elements of 6G [28]:

(1) Spectrum efficiencies 5 to 10 times higher than those of 5G;
(2) Peak data rate of at least 1 Tbps or up to 10 Tbps in some instances that involve THz wireless backhaul and fronthaul (x-haul) [46];
(3) User experienced data rate of 1 Gbps or even up to 10 Gbps in certain cases (e.g., indoor hotspot);
(4) Ten times higher connectivity density than that of 5G which can sustain up to 107 devices per area (in km^2^) and area traffic capacity of up to 1 Gbps per area (in m^2^) for hotspot;
(5) A wireless network that can support various (or at times, conflicting) requirements [47];
(6) High mobility (≥1000 km/h) and over-the-air latency of 10–100 μs for acceptable quality of experience (QoE) in cases that involve hyper-HSR and airline systems;
(7) Huge connection density up to 107 devices/km^2^, which means 10 times the connectivity density of 5G; in addition, area traffic capacity of up to 1 Gbps/m^2^ for scenarios such as hotspots;
(8) 10–100 times higher energy efficiency than in 5G;
(9) 5–10 times greater spectrum efficiency than in 5G.

Table 1 summarizes the differences between 5G and 6G. It shows that 6G will make dramatic changes in mobile communications. Moreover, it could be concluded that 6G will enhance the data rate, latency, spectrum, and connectivity density by up to 10–100 times compared to 5G.

## 5. 6G RF Spectrum Technologies

One of the key features of next-generation mobile communications is the transmitting frequency spectrum. Mobile networks have demonstrated remarkable growth over the years, particularly in terms of spectrum resources (for every generation), given the rising demands for higher speed or data transfer rates. As the development of 6G is expected to offer significantly higher bit rates (up to Tbps), operation at higher frequencies for the available spectrum and bandwidth is expected. Both VLC and THz are spectra with highly promising prospects to realize the vision of 6G [49].

**Figure 3:**
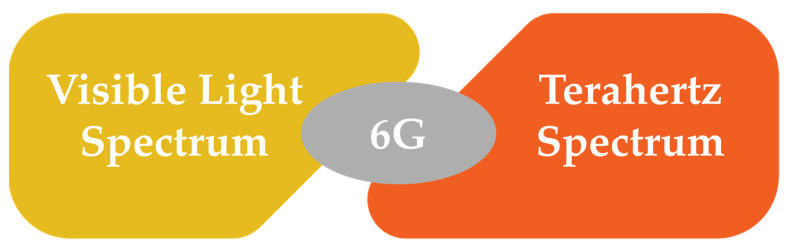
6G spectrum technologies.

Figure 3 presents the two main 6G spectrum technologies, while Table 2 summarizes the different technologies of spectrum communication for 5G and 6G, including mmWaves, THz, and VLC.

### 5.1. THz Spectrum

**Figure 4:**
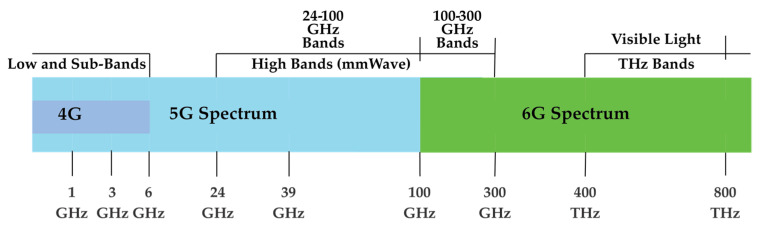
4G, 5G, and 6G spectrum bands.

THz waves are electromagnetic waves with a spectrum that ranges from 0.1 to 10 THz (licensed spectrum) and a wavelength that ranges from 30 to 3000 microns. The spectrum falls within the transition region between macro-electronics and micro-photonics, specifically between microwave (from its lower band) and infrared light (up to its higher band) [55]. Although the frequency band (which ranges from microwave to optical wave) for THz may not be fully developed, THz communication exhibits the strengths of rich spectrum resources and high transmission rate, which offer highly valuable broadband wireless access for future mobile communications [56]. Additionally, THz communication is linked to an extensive bandwidth range (up to 10 THz). Apart from its capacity to penetrate visually non-transparent objects, THz communication also enables interactions of micro-scale objects (e.g., nanonetwork) due to its scale-down antennas (*λ* of about 1 mm for 300 GHz). Figure 4 illustrates the spectrum bands for the generations of 4G to 6G, including mmWaves, THz, and VLC bands.

### 5.2. Visible Light Spectrum

Using infrared, visible light, and ultraviolet spectra, optical wireless communications (OWC) are seen as a complement to RF-based mobile communications. In OWC, visible light with a spectrum that ranges from 430 to 790 THz (unlicensed spectrum) demonstrates the highest potentials, due to the extensive use of light-emitting diodes (LEDs) and technological advancements. In the case of LED, unlike former illumination technologies, its light intensity levels can swiftly change, allowing different ways to encode data in emitted light [57]. VLC makes the most of LED for both lighting and high-speed data communication purposes. Unlike the typical RF bands, VLC presents ultra-high bandwidth (THz) and frequency reuse and unrestricted access to spectrum without electromagnetic interference [58]. The use of visible spectrum and VLC technologies (for short-range links) is necessary to achieve optical fiber performance for the 6G mobile network. However, the current VLC indoor technology today is fixed within a range of a few tenths of a megabit per second to 100 Mbps over 5 m. Nevertheless, the launch of emerging new light sources grounded by micro-LED technology (given the absence of beamforming and commercial off-the-shelf light fixtures) can address such restrictions. Using a single-diode LED in the laboratory, such innovation can provide almost 10 Gbps and a bandwidth of over 1 GHz.

## 6. Enablers for 6G and Relevant Technical Preparation and Implementation

**Figure 5:**
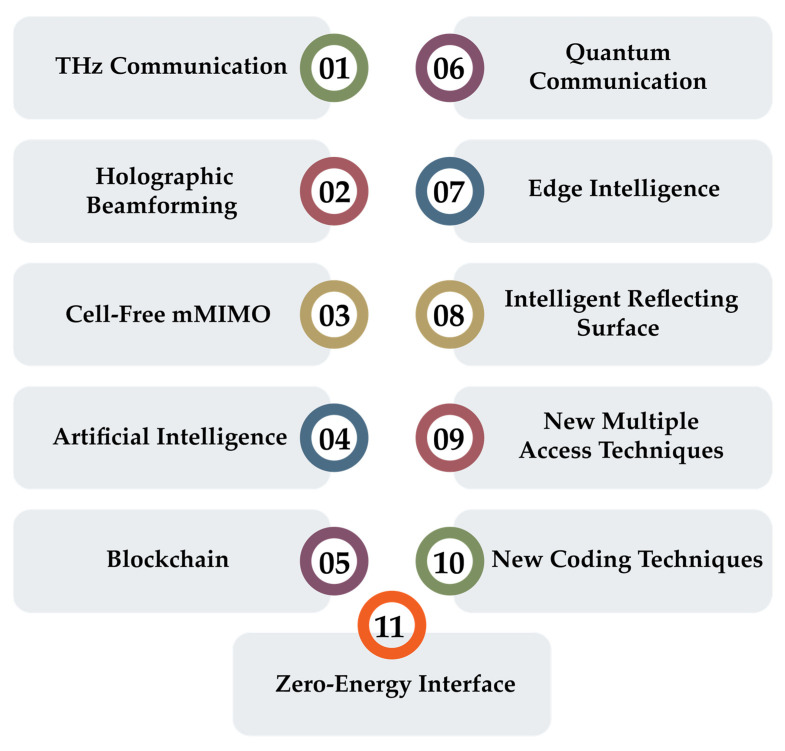
6G key enabling technologies.

Many technologies, such as THz communication, holographic beamforming (HBF), mMIMO, AI, blockchain, and quantum communication (QC), are among the top technologies as 6G key enabling technologies. These technologies are discussed in the following subsection. Figure 5 introduces the most important 6G key enabling technologies.

### 6.1. THz Communication

As the first wireless communication system with ultra-high-speed communication, the use of frequency bands of more than 0.1 THz is required to support features that demand data transfer rates in the unit of Tbps. The capacity of the RF band is inadequate to cater to the rapidly increasing global demand for wireless communication technology, which has propelled the growing interest in THz communication [59] for the 6G network. The massive bandwidth resources in the THz region (which ranges from 0.1 THz to 10 THz) address the limitations of certain technologies in coping with high speed. Through the use of the THz band, the use of 6G can be optimized with significantly improved bandwidth, capacity, speed, and network security. Moreover, the THz band can keep up with the capacity of cells at nanoscale up to micrometer over 10 m without compromising the speed in communication [60] and supporting the Internet of nano-things (IoNT) [61,62].

### 6.2. Holographic Beamforming

**Figure 6:**
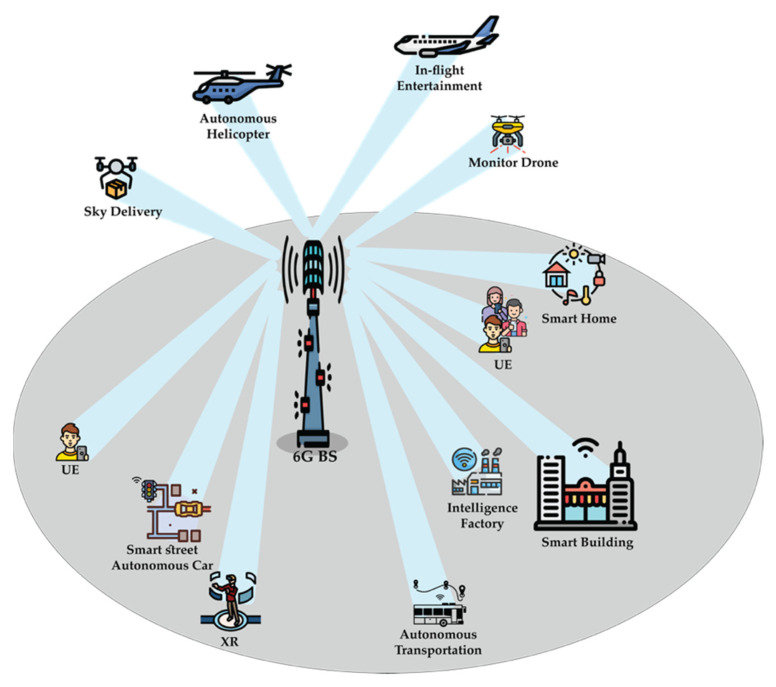
Holographic beamforming.

Beamforming [63] is a technique that utilizes a focused narrow beam with a very high gain for transmitting and receiving utilizing antenna arrays. It does this by concentrating the power in a small angular range. Beamforming improves coverage and throughput while also increasing the signal-to-interference-plus-noise ratio (SINR), which may be used to monitor people. HBF, as shown in Figure 6, is a more advanced beamforming technique that makes use of a software-defined antenna (SDA). RF signals from the radio are routed through an optical hologram to the back of the antenna (a holographic plate) before the beam’s shape and direction are adjusted to reflect these tiny elements [64]. Using HBF allows 6G systems and IoT devices to share radio frequencies in a flexible and efficient manner [65,66]. Moreover, HBF increases the position accuracy in 6G networks and IoT up to centimeters [67].

### 6.3. Cell-Free mMIMO (CF-mMIMO) Technology

mMIMO is starting to be deployed in some markets worldwide, but it is still a relatively immature technology that is expected to mature and provide more applications and use cases in 6G. One of the current challenges of broadband connectivity is providing stable/reliable service quality in a coverage area. The utilization of cell-free (distributed) mMIMO is considered one of the potential solutions to improve coverage for the future. In cell-free structures, all distributed mMIMO should appear as one physical co-located mMIMO to form an ultra mMIMO.

**Figure 7:**
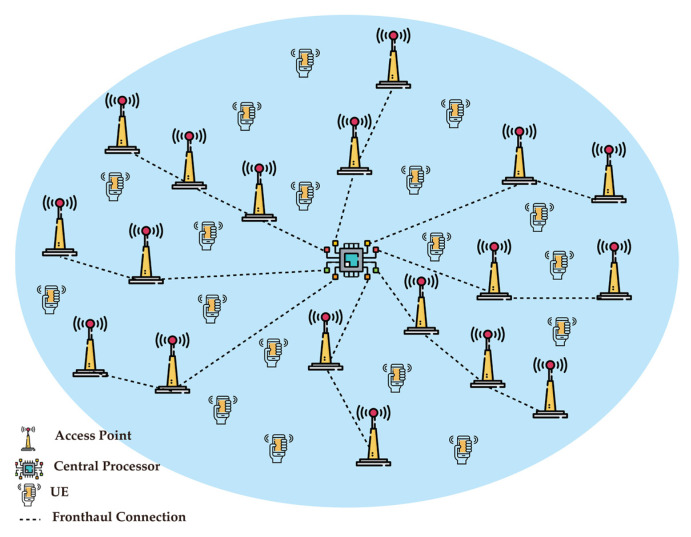
Cell-free massive MIMO.

CF-mMIMO (Figure 7) is considered a spectral and energy-efficient system that is capable of simultaneously serving numerous users over the same radio resource. At the same time, it can effectively reduce radiation power consumption and mitigate interference between adjacent user terminals. However, along with numerous advantages of mMIMO, its performance is limited by challenging problems, such as complex precoding procedure, time synchronization in time division duplex (TDD) [68], and large CSI feedback overhead.

As discussed in Section 4, one of the main limitations of 5G is the issue of time synchronization. In a real-time scenario, multiple objects are going to collaborate with each other in a physical or in a virtual world; therefore, in CF-mMIMO the importance of time synchronization in addition to the CSI overhead and resource allocation will be vital.

### 6.4. Artificial Intelligence (AI)

AI [69] is not part of the earlier generation networks (from 1G to 4G) [64]. It is part of the 5G network, officially from the latest addition in the 3rd Generation Partnership Project (3GPP) Release 18 specifications [35], which is expected to benefit the telecommunications sector with the development of various notable applications [70,71,72,73]. As part of the new 6G network, AI plays an essential role in revolutionizing communication and automation, such as in handover process [74,75,76,77,78], resources allocation [79,80], and network selection. Additionally, integrating AI to the 6G networks leads to higher performance, particularly when delay-sensitive applications are involved. After all, both AI and ML are the key technologies for 6G [49]. Taking the case of ML, its algorithms can be efficiently used for the allocation of BS resources and close-to-optimum performance. Besides that, intelligent materials used in public settings (e.g., buildings or streetlights) can make use of the network with the customized changes (to radio waves). Moreover, deep learning techniques can help to enhance the accuracy of indoor positioning [81].

### 6.5. Blockchain

Blockchain [82,83,84,85] is part of the revolution for future mobile communication technologies. Through the different features of the blockchain (e.g., auditability, decentralization, immutability, and transparency) that are essential for 6G communication, better security features allow secure access to critical data in different network entities and an untamable distributed ledger containing all data is shared among all relevant entities [86]. Besides that, blockchain also offers a number of benefits in resource orchestration and future 6G network access. Apart from cryptocurrency, blockchain is useful for financial and social services, risk management, and healthcare facilities. The role of blockchain in different application domains has been explored in several studies [87]. There are many significant advantages of blockchain-enabled 6G; for example, 6G will be able to monitor and control resource use and sharing more effectively utilizing blockchain [88]. Also, new business prospects, value generation, and competitive advantages can be created on the blockchain in 6G to create sustainable and scalable company models [89].

### 6.6. Quantum Communication

As with the previously described technologies, QC [90], is another critical 6G technology that provides unconstrained communication security. The critical distinction between conventional binary communication and QC is whether on-site eavesdropping is detectable [91]. Due to the use of quantum principles, data encoded in a quantum state (using photons or quantum particles) cannot be accessed or duplicated without altering the data (e.g., correlation of entangled particles and inalienable law). Additionally, the superposition nature of the qubits in QC results in a faster data transmission rate. There are many types of QC at the moment, including quantum key distribution, quantum secret sharing, quantum teleportation, and quantum secure direct communication [92]. A significant advantage of this technology is that it can considerably improve data security and reliability. An adversary’s interference in QC can change one’s quantum state. Because of this, the recipient cannot remain oblivious of the interference [28]. Furthermore, QC considerably improves the network efficiency by providing information in a real-time and ultra-fast response to the user’s demand [93]. Also, QC can improve to provide ultra-low latency in 6G networks. More features that QC provides are [94]:

- relies on qubit;
- accelerates processing data for ML and deep learning models;
- allows for the simultaneous processing of millions of devices;
- allows for more efficient handling of vast amounts of data.

### 6.7. Edge Intelligence

Fifth generation promises to make changes in three different domains: higher speed, design system SDN, and support for edge computing. The latter can have a transformative impact, because, effectively, what edge computing offers is to the end-users of the 5G network. It provides the capability of having systems running locally or at very close edges without going globally. Therefore, it is desirable for end-users to have an environment that could provide a private, secure, and effective connectivity to an edge server. Such a system could facilitate running AI workloads, data analysis, and mining anonymization encryption at a local site [95,96].

One of the earliest adopters of edge computing for the 5G mobile network systems is SK Telecom. They have devised a plan to implement the mobile edge architecture to decentralize the server architecture across South Korea, resulting in reduction of communication delays by up to 60% [97]. This is also part of the efforts to meet one of the “5G triangle”, i.e., URLLC, which is expected to offer close to 1 ms latency.

However, 5G faces some architectural limitations, such as network scalability. One thing that should be taken into account is that the network slices have to be implemented into an individual network router, which possibly leads to certain scalability issues. It is envisioned that in 6G, a new architecture with different approaches for supporting network slices should be available [96].

Another limiting factor that 5G faces is the utilization of mmWave. Although mmWave can provide higher-speed communication, it is limited to short-range communication. In 6G, the trend is toward providing higher speeds via THz communications, further limiting the communication range. Therefore, to provide connectivity for things in an environment, all devices have to receive services under an access point (AP) in their vicinity. Such a type of connectivity is vulnerable to speed mismatch, for example, existence of a very high end on one side of the AP and a lower band and lower speed end on the other side. This leads to the need to enhance edge computing functionality [96].

It is anticipated that 6G, with the smart exploitation of AI methodologies, would drive toward the robust edge capabilities to handle communication mismatch as well as new fundamental architecture to have more intelligent edge computing [98]. Thus, it is expected that existing edge computing technology will evolve into edge intelligence technology by 2030.

### 6.8. Intelligent Reflecting Surface (IRS)

IRS (intelligent reflecting surface), a new concept in wireless communication, is expected to be an extension of mMIMO [99]. It has been considered in recent times as a promising technology that is highly capable of decreasing the energy utilization of wireless networks and also of attaining theoretically unmatched and mMIMO increments [100,101]. IRS involves a large surface area that is placed over a wall or a flat surface. It comprises a significant number of low-cost reflecting electronic components that have reconfigurable variables, such as conventional reflect-arrays as well as varactor diodes or micro-electrical–mechanical systems, the resonant frequency of which can be regulated electronically.

**Figure 8:**
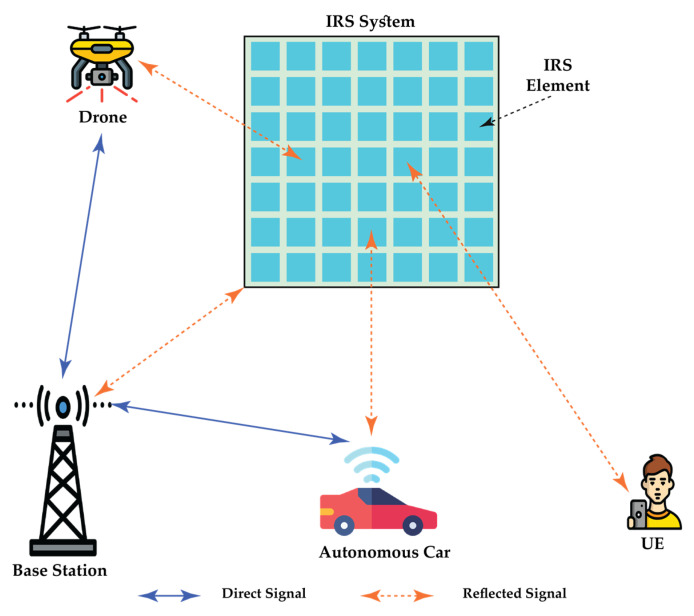
Intelligent Reflecting Surface.

Since IRS is comparatively simple to install and uses low energy, IRS-assisted communications may be used to bring about a significant increase in the coverage of 6G networks in various possible situations [102,103,104]. For instance, IRS can be used to coat the external wall of buildings in the outdoor urban environment. This provides an opportunity to improve the coverage and enhance the energy efficiency of 6G networks in cities. IRS can particularly be used on the surface of high buildings, which produces a virtual line-of-sight (LoS) link among the AP and IRS, which is especially favorable when the direct path is extremely shadowed [105]. In addition, because of the extensive deployment of IRS, there may be a significant decrease in the electromagnetic radiation produced by network infrastructures within the city. IRS can also be used to coat the interior walls in the indoor environment to improve the local connectivity of different types of devices, such as mobile phones and tablets, that depend on wireless connectivity for their operation. IRS can be specifically used in ceilings and underground walls to offer the required connectivity to several users simultaneously [102]. Figure 8 provides an IRS illustration.

### 6.9. New Multiple Access Techniques

The previous generations of cellular networks have adopted radically different multiple access techniques with one common theme in mind: to have orthogonal signals for different users at the receiver side. In view of emerging applications and in order to fulfil the need for massive numbers of connections with diverse requirements in terms of latency and throughput, 6G networks are experiencing a paradigm shift in design philosophy, shifting from orthogonal multiple access (OMA) to non-orthogonal multiple access (NOMA) design. NOMA superposes users at the same time-frequency resource and distinguishes them in the power domain. Compared to the conventional OMA schemes, NOMA allows users to simultaneously share the same resource blocks that are beneficial for supporting many connections in spectrally efficient communications. To date, a variety of NOMA technologies, including power domain NOMA (PD-NOMA), pattern division multiple access (PDMA), sparse code multiple access (SCMA), multi-user shared access (MUSA), and so on, have been proposed [106]. Recently, rate-splitting multiple access (RSMA) [107] has emerged as a powerful and robust non-orthogonal transmission technique and interference management strategy for cellular networks.

In today’s wireless networks, access points commonly employ more than one antenna, which opens the door to multi-antenna processing. The key building block of the downlink of multi-antenna networks is the multi-antenna (Gaussian) broadcast channel (BC). NOMA was initially designed for a single antenna (single-input single-output; SISO) BC. Contrary to the SISO BC that is degraded and where users can be ordered based on their channel strengths, the multi-antenna BC is non-degraded, and users cannot be ordered based on their channel strengths. This is the reason that superposition coding NOMA is not capacity-achieving [108]. RSMA is a more general and more powerful multiple access scheme that relies on linearly precoded rate-splitting with successive interference cancellation (SIC) to decode part of the interference and treat the remaining part of the interference as noise. This provides an opportunity for rate and QoS enhancements and complexity reduction. However, the information and communication theoretic analysis in specific scenarios, to understand benefits in terms of spectral efficiency, reliability, and CSI feedback overhead reduction over conventional strategies used in 5G, is still lacking at the moment.

**Figure 9:**
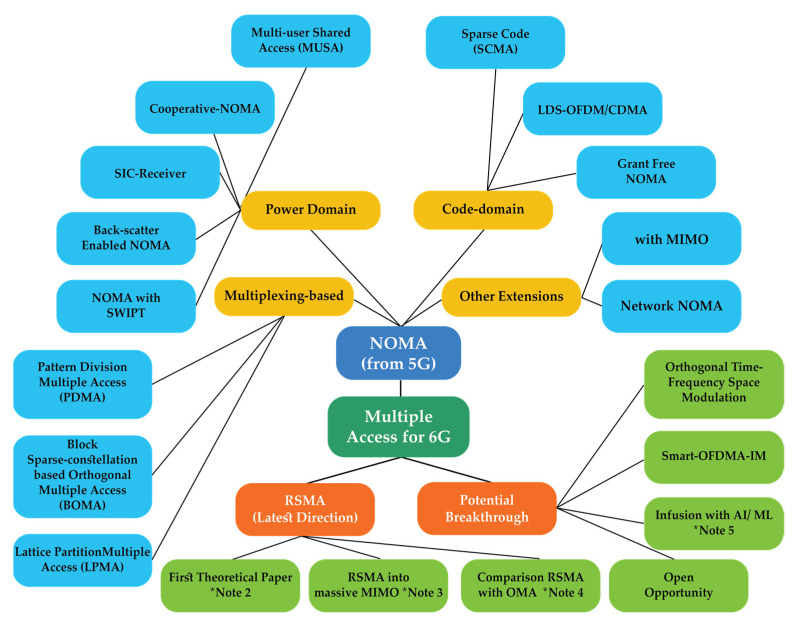
Classification of the potential multiple access techniques for 6G. * Note 2: Ref. [109], * Note 3: Ref. [110], * Note 4: Ref. [111], * Note 5: Ref. [108].

Moreover, despite several emerging proposals focusing on multiple access techniques, there is very little understanding of the performance of these techniques to fulfill 6G requirements. Furthermore, exploitation of potential opportunities to infuse AI/ML into new multiple access techniques has not yet been attempted. At the moment, several multiple access schemes have been devised as candidates for the 6G waveforms. However, recent literature has suggested the RSMA as the most promising multiple access candidate that is expected to fulfill the 6G requirements [109]. Figure 9 presents the classification of the potential 6G multiple access techniques.

### 6.10. New Coding Techniques

With the development of 5G networks, the upgrading of communication technology and devices has been approaching Shannon’s limits. In the traditional Shannon information theory, the correct transmission of information can be achieved based on the forward error correction coding schemes. However, as the number of IoT devices increases, it is very challenging to import massive data into the core network, and network blocking and service quality decline can easily occur. Moreover, the existing modulation and coding techniques are insufficient for the 6G applications operating in the THz band.

Therefore, new coding techniques are crucial for various 6G use cases. For IoT applications that require short packets, the low-density parity check (LDPC) codes and polar codes with short block lengths have been employed for 5G systems for use in traffic and control uplink/downlink channels. However, codes with short block lengths have reduced reliability such that error-free transmission cannot be easily guaranteed [112]. This can lead to an increase in the error probability and a higher requirement for retransmissions, which is not suitable for 6G applications involving ultra-low latency requirements or limited energy capability. Furthermore, low-energy applications are often not suitable for retransmissions, because this requires the device to stay in a non-sleep mode for an extended duration, leading to an increase in energy consumption.

On the other hand, codes with longer block lengths usually result in increased latency. Therefore, new coding strategies should both encompass forward error correction and include novel iterative retransmission/feedback mechanisms and ML-based methods. For long codewords in channels without fading, existing methods, such as turbo decoding and belief propagation, are highly efficient and able to operate close to Shannon’s theoretical limits [113].

In [114], several THz signal coding and modulation techniques, such as high-speed and high-accuracy acquisition and tracking mechanisms, waveform and channel coding, THz direct modulation, THz hybrid modulation, and THz broadcast modulation, have been proposed.

Meanwhile, in [115], a cognitive information transmission based on a 6G “mailbox theory” has been proposed. The mailbox theory requires a smaller amount of data for transmission while achieving higher reliability of decision-making using cognitive information transmission. It is based on the concept of a twin intelligent agent that integrates communication, computing, and caching capabilities. It features self-organization, self-learning, self-adaptation, and continuous evolution capabilities based on information value. The process of interpreting information value is dependent on data attributes such as polarity, dependence, figurability, convergence, dynamics, and traceability.

### 6.11. Zero-Energy Interface

In terms of energy efficiency, the ultimate 6G zero-energy vision can largely be achieved through a combination of efficient hardware design for on-device intelligence, ultra-low-power receiver architecture, enhanced energy harvesting techniques, and zero-energy communication schemes in the massive but scalable machine type communication (MTC) [116]. In this context, significant power reduction can be explored by considering energy consumption of the intelligent device ecosystem as a whole, instead of individual elements with individual antennas and transceivers. Secondly, with the booming energy harvested-based devices coming out, MTC network architecture needs to be advanced to accommodate the development with substantially different requirements. For example, it can complement ambient backscatter communications (AmBC), a promising technology that separates carriers needed to transfer power to the devices and allows backscatter communications. In AmBC, backscatter devices can communicate between one another by modulating and reflecting received RF signals from ambient sources such as waves from nearby RF towers [117]. Most recently, [118,119] developed the complementary security and quality requirement aspects for such schemes for greener communication. The approach consumes significantly less power than the conventional transmitter by replacing the voltage-controlled oscillator and power amplifiers. However, this approach is still challenged with direct path interference, backscatter propagation, co-existence with legacy receivers, and the ambient signal of unknown fast-changing phase and amplitude.

To complement the evolution of hardware for zero-energy, on-device intelligence and zero communication schemes are expected to be fully applied in 6G. The first refers to the application of ML and AI to create context- or application-aware energy management for efficient battery usage, smart compressed sensing, event-driven sensor interfaces, or adaptive communication protocols to optimize data transfer [120]. There is still a lot of ongoing concept development for zero communication schemes, but it can mainly be seen as quality requirement optimization via architecture or device design while considering the PHY to APP layers end-to-end (E2E) aspect, especially with the introduction of THz band, technologies such as CF-mMIMO and IRS, heterogeneous MTC scenarios, and divergent application requirements [121].

## 7. Challenges of 6G

**Figure 10:**
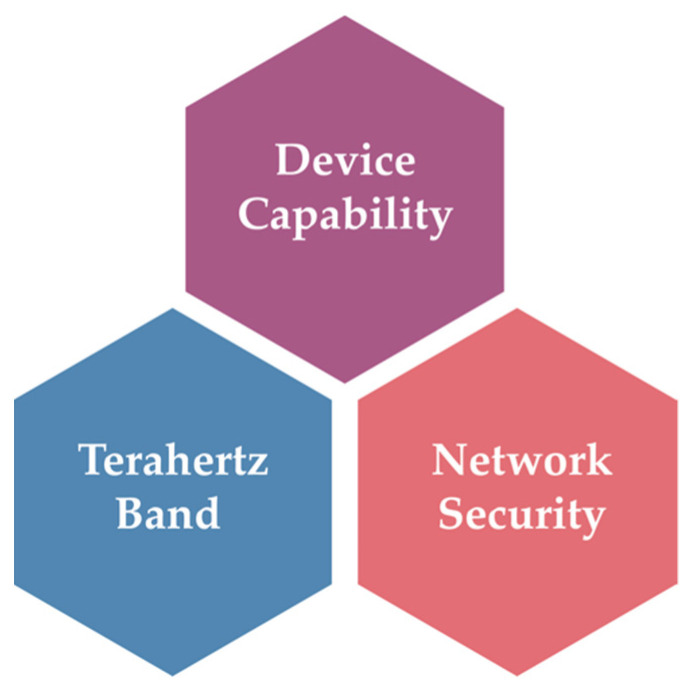
6G prominent challenges.

There are a few technical issues, provided in Figure 10, in the application of the 6G mobile network that should be addressed to satisfy the global technological demands. The following subsections discuss some of the key challenges of 6G.

### 7.1. Device Capability

The technical capabilities of mobile devices should be compatible with the new 6G features, given the extensive use of the 5G mobile network [122]. As mobile devices that support 5G are not compatible with some of the new 6G features, improving the technical capabilities of mobile devices for 6G may contribute to higher costs. However, supporting 1 Tbps throughput, AI, XR, and integrated sensing with communication features using individual devices is challenging. Several other important points that increase the device capability challenges of 6G deployment are:

- Hardware for the research and development of 6G is still not ready. Having equipment and modern labs helps the developers and devices designers to test and develop the devices and produce compatibility with 6G technology.
- The industry standard is still not finalized. There are no standards for the devices that can work with 6G, which complicates things for the device’s manufacturer. Furthermore, organizations such as 3GPP, which provides mobile technology standardization, have still not defined the standards of 6G technology.
- Circuit design for 6G device at THz requires plenty of validation works in the R&D stage. At the current stage, there are plenty of research works focusing on characterizations and modeling at the THz level, especially the first work on THz [123] and those from NYU Wireless [124].

### 7.2. THz Band

To address the demand for future applications, the need for higher data rates, and lower latency, each generation of cellular system needs to open up new bands. Based on the laws of physics, the spectrum beyond the mmWave looks suitable for short-range communication and ambient sensing [37]. Unlike the mmWave, there is a large amount of spectrum available in the frequency range of 90 to 120 GHz, which is also known as low terahertz. Although THz band refers to the frequency range of 0.1–10 THz [125,126], in literature, the term THz spectrum refers to the frequency range somewhere between 100 GHz and 750 GHz, which is considered as upper mmWaves that are currently used in 5G applications [127,128].

Although the high frequencies permit high-speed data rates, the downside is the higher the frequency, the higher the signal attenuation and the shorter the communication range. However, the use of directional antennas in mmWave started with 5G, and it was shown that by properly designing a directional antenna, the communication distance can be enhanced. On the other hand, in 6G, it is necessary to make a tradeoff between data rate, communication distance, and the targeted use cases/applications.

The THz is not a new frequency band; it has already been used over the past decades, for example, in medical devices and satellite radio communications. However, bringing the THz range to cellular communication is new, and different kinds of tradeoffs need to be carried out to adapt this frequency band to new use cases.

On the one hand, the utilization of higher frequency bands is limited by the speed of the state-of-the-art technologies, and yet there is no cost-effective equipment for commercial usages. As THz band has been used for different applications, transistor technologies can make amplifiers up to the 1 THz range. However, combining and packaging that together with the mainstream CMOS for digital signal processing requires many steps that are advanced both in terms of technology and science [37]. Therefore, new hardware components are required, for example, for power amplification, sampling, and processing.

**Figure 11:**
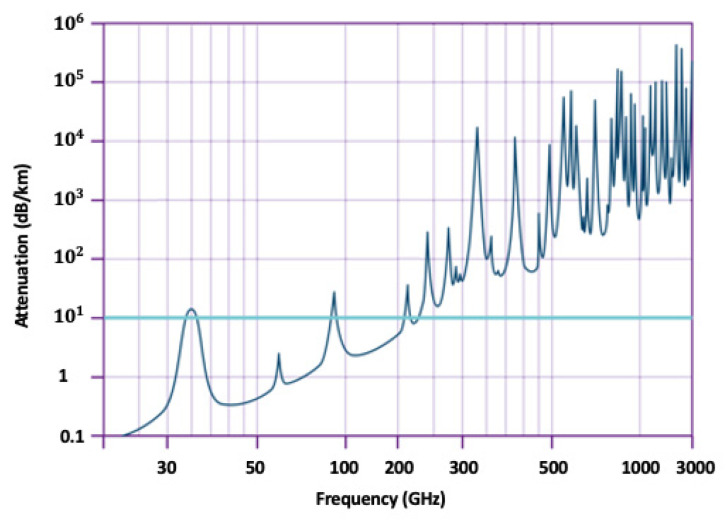
Atmospheric attenuation of radio waves from 30 GHz to 3 THz [130].

On the other hand, long-distance communications are linked to significantly higher atmospheric absorption and propagation losses, as can be seen from Figure 11. In order to address atmospheric losses alone, the first characteristics of propagation in the THz frequency range should be identified; then, transceivers should be designed accordingly to support high-frequency band, a wide range of bandwidth, power, sensitivity, and low noise figures. Furthermore, new multipath channel models must be formed to address the frequency dispersion issue, given the wide range of bandwidth needed [129]. The currently available modulation and coding methods are also inadequate to support the THz band, while new method development is complex and challenging. Thanks to high penetration loss of THz band and consequently short-range communication, the interference issue will be limited to a shorter range, i.e., interference needs to be mitigated more locally, compared to the existing interference cancellation techniques in 4G and 5G. However, due to the trend of unitization of wider bandwidth and adaptive frequency range, the problem of interference with other coexistent technologies (THz use cases) is anticipated to be critical and should be effectively addressed before the realization of 6G.

In order to provide impactful research and innovation in THz communication, first it is required to conduct a study on channel behavior and propagation characteristics of THz communications. On the other hand, it is necessary to know what parts of the THz bandwidth spectra need to be focused on for future communication and how much bandwidth is available in that frequency range.

In addition, from the perspective of hardware development, providing high-speed communications, like terabits per second, is highly challenging, because in this frequency range, the electronic circuitry needs to be moved to more optical circuitry, and a large amount of bandwidth will be required. There are also health and safety concerns in the utilization of the high-frequency range, in which, for the safety of users, the power constraint should be considered and applied to the devices.

### 7.3. Network Security

As 6G would be extensively used to connect numerous mobile devices, smart devices in automation, AI, XR, satellites, and smart cities, the capacity of security techniques for 5G would be inadequate for 6G. To establish reliable network security for 6G, innovative cryptographic techniques, integrated network security techniques, and physical layer security techniques are necessary [3], particularly at lower cost and complexity without compromising network security.

## 8. Future Directions in 6G Technology Development

**Figure 12:**
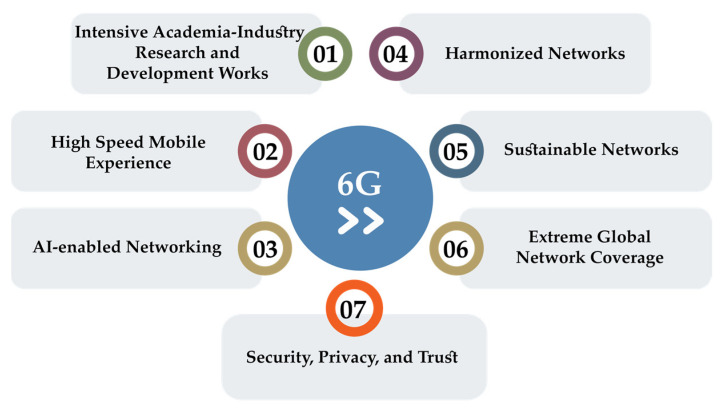
Future trends and directions in 6G technology development.

The current vision and concept of 6G give a comprehensive view of future research paths and significant obstacles in implementing 6G mobile networks. The future research directions related to 6G (Figure 12) are described in the following subsections.

### 8.1. Intensive Academia–Industry Research and Development Works

Although 5G has not reached its full commercial deployment, some nations have developed particular policies to create 6G in recent years [3]. In Finland, an eight-year initiative with a budget of USD 290 million, dubbed the 6Genesis Flagship program, was initiated in 2018 to build a 6G ecosystem [131]. Aside from the United Kingdom and Germany, which are investing in 6G technologies such as quantum technology, the United States has begun investigating THz-based 6G mobile networks, while China has stated its intention to create 6G. The development of 6G has also piqued the interest of several businesses, including a large test and measurement vendor, Keysight Technologies, with the potential to dominate the worldwide market in 6G technologies, services, and solutions. Several large corporations, including Ericsson, Huawei, Nokia, and Samsung, have stated their commitment to 6G research and development [132,133]. Furthermore, Ericsson, Nokia, and SK Telecom have formed a strategic partnership in 6G research [134].

Another collaboration is between the Massachusetts Institute of Technology (MIT) and Ericsson [135]. MIT and Ericsson aim to conduct two research projects which focus on the pattern of state-of-the-art hardware to power the 5G and 6G mobile networks. The speed, computation power, and energy efficiency of cognitive networks are the main topics of the two research projects. The first project will explore essential studies on the computer materials and chips based on lithium-ion that replicate the human brain’s structure in order to consume only a fraction of the energy of today’s silicon-based chip designs. The second project focuses on discovering the methods, such as energy harvesting from radio signals and other sources, and undemanding power system designs to accomplish simple tasks, such as autonomous connection and control with minimal to zero need for charging.

### 8.2. High-Speed Mobile Experience

The 6G wireless networks are expected to yield higher data rates, approximated to Tbps, which vary with the type of technology employed. Such technologies may include THz communication, high beamforming, better coding methods, and channel access plans. This area of 6G KPIs needs to be explored in detail. The data preparation and storage facility required for connecting future 6G wireless networks to edge intelligence devices can be achieved by integrating cloud/edge/mist platforms with wireless platforms. In addition, the metric of quality of physical experience (QoPE) supports the linking of AI technology-inferred human insights, QoS, and QoE, which offers support in various ways. In this regard, it is imperative to mention that QoPE supports XR and holographic communication to allow the human brain to practically sense any experience. With the introduction and use of 6G networks and technology, the current network security and protection measures will also be enhanced. Thus, efforts are being invested in integrating new techniques, such as secure channel coding, channel-based adaptation, quantum key distribution, and quantum teleportation, with future 6G wireless networks [136].

### 8.3. AI-Enabled Networking

It is expected that the world of communication will benefit from the potential 6G wireless networks in the form of worldwide coverage and services with variable levels of QoS. Sixth generation networks equip the whole network with storage and computing capabilities spread from the central part of the cloud to its edge or to the remote device at the client’s end. The principal role of the AI technique in hyper-intelligent 6G networking is quite apparent. The fundamental networking processes can be understood by conducting more investigations on logical AI solutions. Hybrid centralized-distributed AI configurations support better utilization of the information gathered from IoT devices and the processing capability of these devices. It is also feasible to prepare a learning model through the worldwide server at central clouds and satellites. However, it must be noted that data transfer from IoT devices to a central server in one go may not be possible at times. Hence, progressive, hierarchical data transfer is adopted whereby data are transferred from end devices to edge nodes and global nodes.

### 8.4. Harmonized Networks

Future harmonization is another important area to be considered during prospective 6G research. Harmonization involves incorporating nanonetworks, nodes (requiring frequencies higher than 100 GHz), self-running D2D, and CF-MIMO. Better radio access visualization techniques can be used to achieve this harmonization in 6G research, since these techniques support programmability as well as abstraction, leading to network harmonization. Network harmonization is one of the most anticipated features of the advanced service-based 6G networks and must be attributed to adaptability, re-ease of use, and optimal assistance independence. This is only possible when 6G is equipped with something beyond the traditional cloud-based network capacities of 5G. In other words, 6G is expected to offer server-less architectures or other such computing paradigms by modifying the legacy mobile architectures in terms of signaling techniques and interfaces. Network harmonization must also offer high reliability, accountability, availability, and liability to ensure proper execution. All the above-mentioned features can be incorporated in network harmonization through communication–control co-design [136,137] and spatiotemporal network design [138,139] or other cross-layer techniques as well as computing techniques such as blockchain [140]. Moreover, forecasting trajectory, resource, and spectrum usage is also found to yield better harmonization results.

### 8.5. Sustainable Networks

The need of the hour in this fast-moving world is to adopt sustainable practices in communication systems on a large scale. Verma et al. [141] put forward the idea of supporting 6G communication through the employment of hybrid whale spotted hyena optimization; this practice allows better cluster heads selection. Moreover, the offloading technique is also being used with the help of combined and transfer learning to delegate part of centralized services to edge computing devices in the vicinity. This was preceded by the introduction of the scheduling concept in 5G and 6G network BSs in Release 15 [142] and later enhanced in Release 16 [33]. This practice allows proper planning of BSs’ sleep and wake schedule to optimize the energy requirement. Wang et al. [143] suggested that the wake-up schedule for BSs can be planned with the help of hybrid energy-powered cellular networks, leading to improved energy efficiency and modified network resources. Network load switching, balancing, and route strategies can be selected in the same way. Transmissions energy is also optimized by integrating AI optimization techniques into 6G networks. Charef et al. [144] proposed duty cycles scheduling performance enhancement using hybrid ML models with the aim to achieve higher QoS performance for massive and 3D MTCs.

### 8.6. Extreme Global Network Coverage

Optimized 3D localization, along with network planning, is essential during the installation of flying relays or BSs. Unmanned aerial vehicles (UAVs) or drones are attributed with resource and capacity limitations and involve heterogeneous processing, thus making it essential to conduct such research. Other important concerns regarding the worldwide network coverage that must be investigated and considered in future research include the use of technology for ensuring smart 3D roaming mechanisms for heterogeneous networks and also ensuring that various operators support secure and safe interoperability. Future research must evaluate the FL (fuzzy logic)-based optimization, which involves local training of ML models by different networks. The networks in FL-based optimization involve update sharing on models instead of information sharing, thus leading to a better 6G ecosystem across the world and ensuring universal network coverage at the same time. Moreover, channel estimation and dynamic link optimization have also been identified as popular subjects for future research leading to extensive global coverage. More effective and reasonable global coverage solutions can be developed by implementing complex procedures.

### 8.7. Security, Privacy, and Trust

The 5G and 6G services and technologies are expected to take the world by storm in the near future, offering better services and technologies to end-clients, communications, businesses, governments, etc. Such developments will develop an enormous attack vector attributed with high risk towards businesses and individuals. Nevertheless, the proper execution of 6G networks seems difficult considering the outdated security techniques, privacy, integrity, availability, and authenticity in 4/5G. However, better execution of 6G technologies is possible by integrating a security by-plan to meet the evolving and complex needs associated with data privacy, location privacy, identity privacy, and meta-data privacy. In conclusion, we can infer that future research in the domain of 6G must focus on the development of better and cheaper security and protection features [145].

## 9. Conclusions

We are currently at the beginning of the early 5G commercial stage, which is touted as causing a substantial revolution—or, at least, evolution—in the mobile wireless communications industry. The revolution aspect of 5G concerns how it “stretches” the use cases that previously focused on mobile broadband only into additional use cases: ultra-reliable low-latency communications and massive machine-type communications. Thus, the term “5G triangle” was coined by the mobile communications industry.

Although 5G just launched in some countries and regions and is mostly still in its infancy, researchers and developers are already conducting their studies and experiments on the development of 6G, especially related to new communication techniques and technologies. Sixth generation is the upcoming mobile system which is expected to be released in 2030 and will significantly change in future mobile wireless networks. From this review, it can be observed that the “5G triangle” will evolve towards the “6G hexagon” by providing additional dimensions to unlock new industrial use cases. Moreover, 6G will provide an extreme data rate and ultra-low latency, which are forecasted at up to 10 Tbps and 10–100 μs, respectively. Additionally, it will enhance spectrum efficiency and connection density to 10–100 times higher than 5G. Furthermore, 6G will bring new applications beyond IoE, enabling intelligent IoE and the vision of everything being connected 100% intelligently. Sixth generation is the era of seamless machine-to-human interactions, things intelligence, and unification of the virtual and physical world. The ultra-high reliability, ultra-high flexibility, ultra-high level of privacy and security, and ubiquitous coverage are the primary keys of the 6G vision.

This paper provides a comprehensive review of 6G technology, including its vision, use cases and applications, technical requirements, and frequency spectra. Additionally, it discusses the key 6G enabler technologies and the crucial challenges that need to be addressed to realize the 6G commercial systems. Moreover, a comparison between 5G and 6G in terms of the most important KPIs is provided. Finally, this review presents the future directions in 6G technology.

## Figures and Tables

**Table 1:** Comparison of 5G and 6G communication techniques [28,40,48,49,50,51,52,53,54].

Characteristic	5G	6G
Operating frequency	3 GHz–300 GHz	Up to 1 THz
Peak data rate	20 Gbps	1 Tbps
Latency	1 ms	10–100 μs
Mobility	500 km/h	>1000 km/h
Available spectrum	30 GHz	10–100 times higher than 5G
Spectral efficiency	30 bps/Hz	100 bps/Hz
Energy efficiency	High	Ultra-high
Connection density	106 devices/km^2^	107 devices/km^2^
Coverage	99.99%	99.9999%
Positioning precision	Meter precision	Centimeter precision
Satellite integration	Partial	Fully
Automation integration	Partial	Fully
Network awareness	Partial intelligibility	Ubiquitous intelligence
Reliability	1–10^−5^	1–10^−9^
Service level	VR/AR/3D	Tactile
XR	Partial	Fully
Haptic communication	Partial	Fully
Smart city components	Separated	Integrated
IRS	-	Yes
Standards	5G/NR	-
Core network	IoT	IoE
HetNets	Flexible	Ultra-flexible
Usage scenarios	EMBB, URLLC & mMTC	uMUB, uHSLLC & uHDD
Main technologies	mmWave, mMIMO, UDN, SDN	THz, SM-MIMO, Laser and VLC, Quantum, Blockchain, AI/ML
Applications	VR/AR/360° videos, UHD videos, V2X, IoT, Smart city/factory/home, telemedicine, and wearable devices	Holographic, tactile/haptic internet, full-sensory and reality, fully automated driving, industrial internet, space travel, deep-sea sightseeing, and Internet of bio-nano-things
Flexible spectrum	Flexible duplex	Free duplex

**Table 2:** Comparison of 5G (mmWaves) and 6G (THz and VLC) technologies.

	5G Technology	6G Technology	
	mmWaves	THz	VLC
Frequency band	3 GHz–99 GHz	100 GHz–10 THz	430 THz–790THz
Supporting data rate	Gigabits/second	Terabits/second	Gigabits/second
Propagation loss	Low propagation loss (compared to THz)	High propagation loss	High
Underwater communication	No	No	Yes
Link	NLOS	NLOS	LOS
Spectrum	Licensed	Licensed	Unlicensed
Electromagnetic interference	Yes	Yes	No
Penetrate through opaque objects	Yes	Yes	No
Challenges	Circuit design, High propagation loss	Molecular absorption, circuit design challenges, higher propagation loss (compared to mmWave), high penetration loss	Small coverage, require RF uplink, dark objects absorb light waves, suffer from shot noise caused by another light source
Environment communication	Indoor/outdoor	Indoor/outdoor	Mostly indoor
Potential	Wide bandwidth, small antenna size, focused beams, path loss can be compensated, and allowing spatial multiplexing	High bandwidth (100x mmWave), small antenna size, focused beams	Low-cost hardware, low interference, unlicensed spectrum
Potential applications	Small cell access, cellular access, and wireless backhaul	Autonomous vehicles, cloud, mobile HetNets	Li-Fi, visible light ID system, hospital robots, underwater communication, and traffic communication systems
Transmission power	High	High	Low

## Data Availability

Not applicable.
